# GWAS unveils features between early- and late-flowering pearl millets

**DOI:** 10.1186/s12864-020-07198-2

**Published:** 2020-11-10

**Authors:** Oumar Diack, Ghislain Kanfany, Mame Codou Gueye, Ousmane Sy, Amadou Fofana, Hamidou Tall, Desalegn D. Serba, Leila Zekraoui, Cécile Berthouly-Salazar, Yves Vigouroux, Diaga Diouf, Ndjido Ardo Kane

**Affiliations:** 1 Centre d’Étude Régional pour l’Amélioration de l’Adaptation à la Sécheresse, Institut Sénégalais de Recherches Agricoles, Thiès, Sénégal; 2 Laboratoire mixte international Adaptation des Plantes et microorganismes associés aux Stress Environnementaux, Bel-Air, Dakar, Sénégal; 3 Centre National de Recherches Agronomiques de Bambey, Institut Sénégalais de Recherches Agricoles, Bambey, Sénégal; 4 Centre de Recherches Zootechniques de Kolda, Institut Sénégalais de Recherches Agricoles, Kolda, Sénégal; 5 Agricultural Research Center-Hays, Kansas State University, Hays, Kansas USA; 6 Unité Mixte de Recherche DIADE, Institut de Recherche pour le Développement, Montpellier, 911 Avenue Agropolis cedex 5, 34394 Montpellier, France; 7 Laboratoire Campus de Biotechnologies Végétales, Faculté des Sciences et Techniques, Université Cheikh Anta Diop de Dakar, Dakar-Fann, Code postal 10700 Dakar, Sénégal

**Keywords:** Senegal, Pearl millet, Morphotypes, Flowering, Diversity, GWAS

## Abstract

**Background:**

Pearl millet, a nutritious food for around 100 million people in Africa and India, displays extensive genetic diversity and a high degree of admixture with wild relatives. Two major morphotypes can be distinguished in Senegal: early-flowering Souna and late-flowering Sanio. Phenotypic variabilities related to flowering time play an important role in the adaptation of pearl millet to climate variability. A better understanding of the genetic makeup of these variabilities would make it possible to breed pearl millet to suit regions with different climates. The aim of this study was to characterize the genetic basis of these phenotypic differences.

**Results:**

We defined a core collection that captures most of the diversity of cultivated pearl millets in Senegal and includes 60 early-flowering Souna and 31 late-flowering Sanio morphotypes. Sixteen agro-morphological traits were evaluated in the panel in the 2016 and 2017 rainy seasons. Phenological and phenotypic traits related with yield, flowering time, and biomass helped differentiate early- and late-flowering morphotypes. Further, using genotyping-by-sequencing (GBS), 21,663 single nucleotide polymorphisms (SNPs) markers with more than 5% of minor allele frequencies were discovered. Sparse non-negative matrix factorization (sNMF) analysis confirmed the genetic structure in two gene pools associated with differences in flowering time. Two chromosomal regions on linkage groups (LG 3) (~ 89.7 Mb) and (LG 6) (~ 68.1 Mb) differentiated two clusters among the early-flowering Souna. A genome-wide association study (GWAS) was used to link phenotypic variation to the SNPs, and 18 genes were linked to flowering time, plant height, tillering, and biomass (*P*-value < 2.3E-06).

**Conclusions:**

The diversity of early- and late-flowering pearl millet morphotypes in Senegal was captured using a heuristic approach. Key phenological and phenotypic traits, SNPs, and candidate genes underlying flowering time, tillering, biomass yield and plant height of pearl millet were identified. Chromosome rearrangements in LG3 and LG6 were inferred as a source of variation in early-flowering morphotypes. Using candidate genes underlying these features between pearl millet morphotypes will be of paramount importance in breeding for resilience to climatic variability.

**Supplementary Information:**

The online version contains supplementary material available at 10.1186/s12864-020-07198-2.

## Background

Pearl millet [*Pennisetum glaucum* (L.) R. Br., syn *Cenchrus americanus*] is an integral part of the diet of many people, particularly in Africa and Asia. Pearl millet grain has a highly nutritional composition (high protein and fiber contents, richer in energy and essential minerals like iron and zinc) than other cereals [1]. Gluten free with a low glycemic index and hypoallergenic properties, pearl millet could therefore be promoted as nutrient-rich food to promote health and enhance food security [2].

Pearl millet is an annual C4 plant that is cultivated in the driest environments. Due to its extensive genetic diversity combined with a high degree of admixture with wild relatives, pearl millet displays wide morphological and genetic diversity [3, 4]. For example, late-flowering morphotypes are often more sensitive to photoperiod than early-flowering morphotypes [5]. Genetic studies already identified polymorphisms among *P. glaucum PHYTOCHROME C* (*PgPhyC*) and *MADS11* (*PgMADS11*) genes associated with yield and early flowering time [6–8]. Such variants would be useful in breeding strategies to cope with recurrent drought periods that have been widespread in the west Sahel in the last three decades. Indeed, flowering time is a trait that plays an important role in pearl millet adaptation to climate variability since its synchronization with the rainy period enables optimal development of the crop. A better understanding of the genetic features of flowering time would be useful to breed pearl millet optimized for cultivation in different climatic areas.

In Senegal, two major varieties of pearl millet are cultivated: Souna morphotypes that flowers early, from 50 to 60 days after planting, and Sanio morphotype that flowers late, from 80 to 110 days after planting [9, 10]. Between 1992 and 2014, a collection of landraces was established that captured nationwide cultivated pearl millet landraces in Senegal. Using agro-morphological traits, microsatellites, and single nucleotide polymorphism markers, previous studies identified genetic differentiation between the two morphotypes [8, 9, 11]. The genetic structure of pearl millet morphotypes in Senegal correlates with a north-south rainfall gradient [8] with little geographic structure [11]. However, we still do not know which genomic regions are associated with the major difference in flowering time and, more broadly, the morphological differences between the two genetic pools.

One way to better characterize the genetic diversity of this large pearl millet collection is to define and use a representative subset of landraces in the panel [12]. Several approaches have already been used to characterize pearl millet diversity using genotyping-by-sequencing (GBS) [11] and even full genome resequencing [13]. The markers developed in such approaches could be used for association studies for adaptation to marginal environmental conditions.

In this study, a core collection of cultivated pearl millets from Senegal was first defined. The landraces were then field evaluated, GBS-sequenced, and used in a genome-wide association study (GWAS) aimed at identifying quantitative trait loci (QTLs) associated with phenological and phenotypic traits between early-flowering (Souna) and late-flowering (Sanio) landraces. Key SNPs as well as candidate genes underlying flowering time, tillering, biomass and plant height of pearl millet were identified. Their use in breeding program to address environmental challenges and farmer’s preferences are discussed.

## Results

### Establishment of a core germplasm collection

**Fig. 1:**
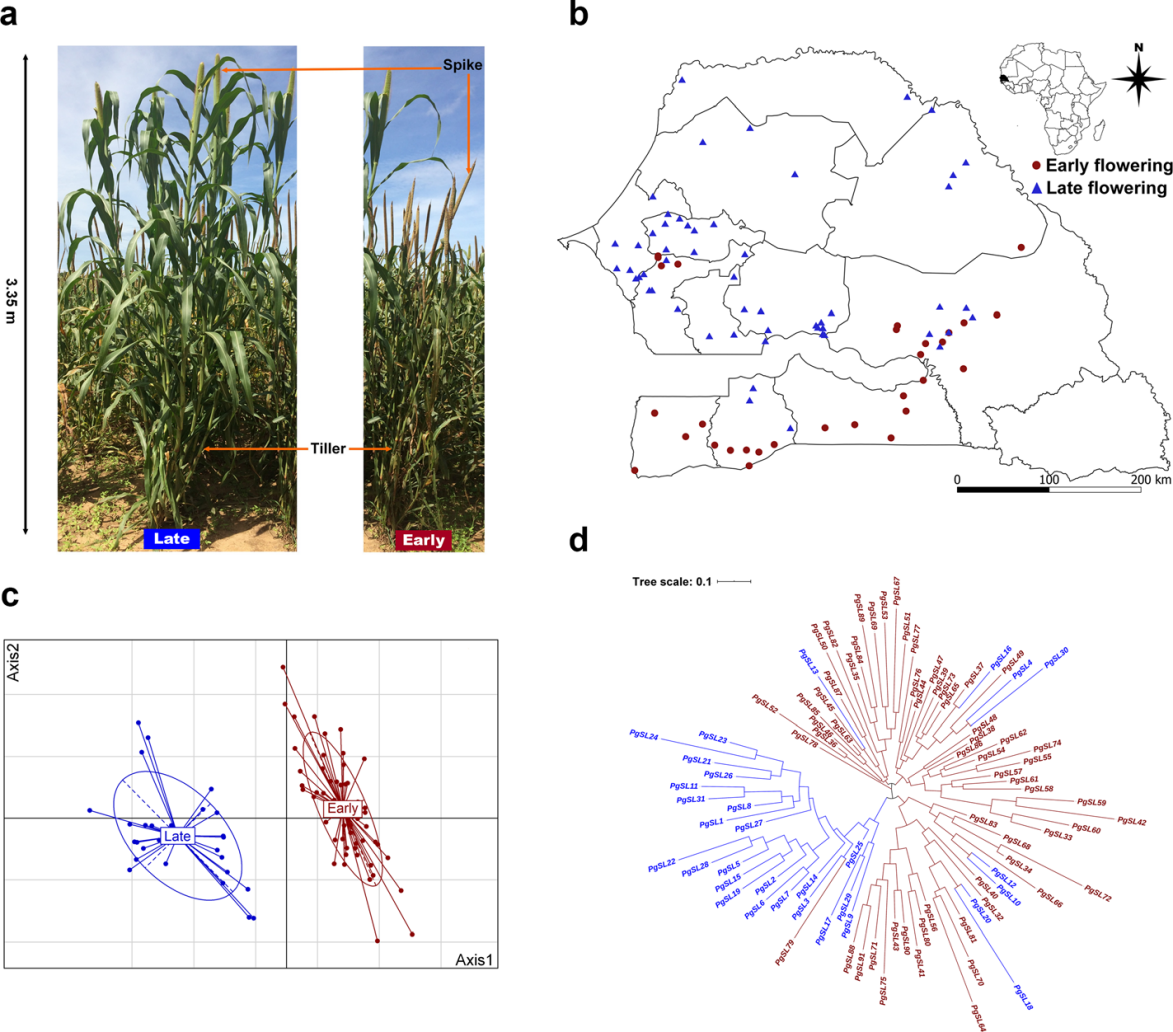
Morphological features, distribution and relationship between cultivated pearl millet in Senegal. **a.** Differences in height, spike and tillering in early-flowering (Souna) and late-flowering (Sanio) morphotypes; **b.** The geographical distribution of 91 landraces [(60 early-flowering (red dots) and 31 late-flowering landraces (blue triangles) capturing the genetic diversity of cultivated pearl millet in Senegal was mapped using QGIS software v 3.8 (https://www.qgis.org); **c.** Principal component analysis (PCA) of early-flowering (red dots) and late-flowering morphotypes (blue triangles); **d**. Neighbor-joining (NJ) tree of the early-flowering (red) and late-flowering landraces (blue)

To capture the diversity of cultivated pearl millet in Senegal, 392 landraces collected nationwide were analyzed. Early- and late-flowering morphotypes differed in plant height, tillering, and biomass, heading and flowering time (Fig. 1a). Using a heuristic approach to select individuals among a large population set with a coincidence rate (%CR) and a variable rate (%VR) > 80% and a percentage of mean (%MD) with a percentage of variance difference (%VD) < 20%, a core representation of 91 landraces was established, including 60 early-flowering Souna and 31 late-flowering Sanio. The paznel includes landraces from all areas in Senegal where pearl millet is grown and covers 22.5% of the germplasm collected nationwide (Fig. 1b, Table S1). All statistical consistency criteria (%MD, %VD, and %VR) had high scores (> 90%), except the variance difference percentage in the core collection of early-flowering Souna. The percentage coincidence rate (%CR) was 97% in the early-flowering Souna subset and 92.5% in the late-flowering Sanio subset (Table S2). Likewise, genetic analysis captured mostly existing alleles in the germplasm. The Nei diversity index was 0.62 among the 91 landraces and 0.56 in the total collection. Therefore, reducing the number of landraces did not cause significant loss of diversity (*P*-value = 0.49). Similarly, principal component analysis (Fig. 1c) and neighbor-joining (NJ) analysis based on Nei genetic distances revealed phylogenetic relationships between landraces (Fig. 1d) that support two genetic pools of pearl millet morphotypes.

### Phenotypic traits discriminating early- and late-flowering millets

To identify phenotypic traits that differ in early- and late-flowering millets, field evaluations of 16 traits were conducted at ISRA-Nioro research center in the 2016 and 2017 rainy seasons. Analysis of variance of the data revealed wide phenotypic variability of the 91 landraces for almost all traits, except 1000-seed weight (Table 1). This points to a highly significant effect of genotype on traits. The interactions between genotypes and years were significant to different degrees in all agro-morphological characters, except in the downy mildew damage score (α = 0.05 and *P*-value = 0.984), tillering (α = 0.05 and *P*-value = 0.307) and panicle exertion (α = 0.05 and *P*-value = 0.158) (Table 1).

**Table 1:** Analysis of variance and heritability (*h*^*2*^) of the 16 phenotypic traits in the defined Senegalese pearl millet core collection. DF = degree of freedom, DMI = downy mildew, HTM = 50% heading time, FLO = 50% flowering time, NTN = nodal tillering, ILE = internode length, MSD = main stem diameter, PHE = plant height, NPT = tillering, FLL = flag leaf length, FLW = flag leaf width, SLE = spike length, STH = spike thickness, SWE = 1000 seed weight, PEX = panicle exertion, BMS = biomass, and GYI = grain yield. Significance: * *P*-value < 0.05, ** *P*-value < 0.01, *** *P*-value < 0.001, NS = Not significant

	DF	DM	HT	FLO	NPT	NTN	IL	FLL	FLW	MSD	PH	SL	ST	PE	SW	Biomass	GY
**Genotype effect**	**90**	***	***	***	***	***	***	***	**	NS	***	***	***	***	NS	*	**
**Year effect**	**1**	NS	NS	NS	**	***	NS	*	NS	***	NS	NS	***	NS	***	***	**
**Genotype x Year**		NS	***	***	NS	*	**	*	*	***	***	*	*	NS	***	***	***
**Means**		0.03	56.37	60.68	6.36	9.33	21.11	46.09	4.59	4.19	241.62	48.9	8.43	3.15	5.84	1528.39	1637.49
**Strd deviation**		0.07	12.27	12.68	2.16	2.19	1.93	6.53	0.66	1.6	39.11	9.35	1.21	3.16	1.44	1114.7	1112.15
***h***^***2***^		0.69	0.97	0.98	0.86	0.96	0.68	0.62	0.6	0.23	0.94	0.9	0.84	0.71	0	0.42	0.55

**Fig. 2:**
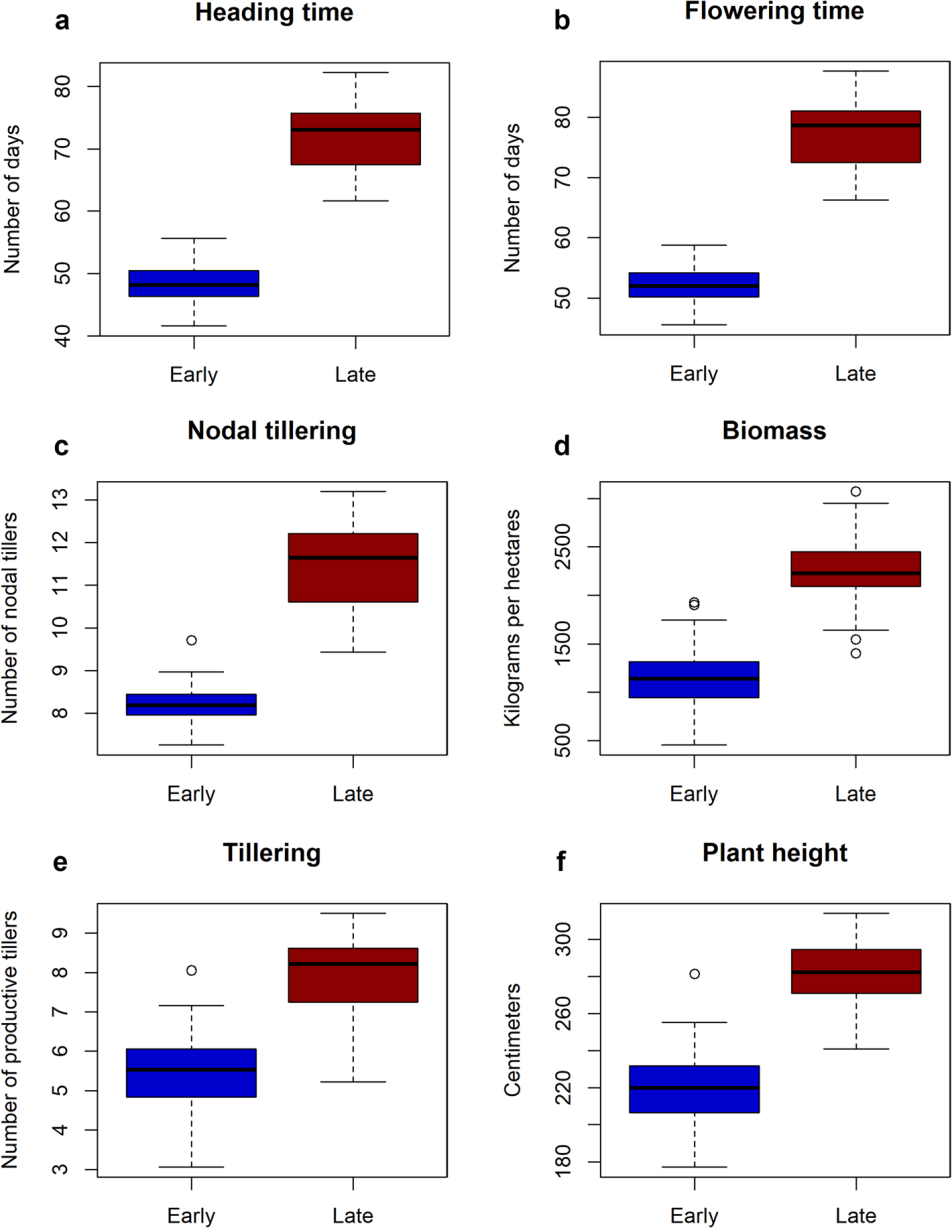
Six traits discriminating early- and late-flowering morphotypes. **a-f.** Boxplots of heading time, flowering time, nodal tillering, biomass, tillering, and plant height, respectively

The first two axes of the principal component analysis of all the phenotypic traits assessed explained 63.7% of total variance and showed the strong agro-morphological structure of early- and late-flowering morphotypes (Fig. 1c). Further, discriminant analysis revealed 12 traits that differed significantly between early- and late-flowering morphotypes (*P*-value < 0.0001) (Table S3). Six traits showed some correlation with the differentiation axis (≥ 0.7) and were thus considered to be highly discriminating characters with high heritability (*h*^*2*^ ≥ 0.5) (Table 1). They can be classified in two types of characters: phenology, which groups heading and flowering time traits, and phenotypic, which groups biomass, plant height, and tillering traits (Fig. 2).

### Genomic variation of the population

**Fig. 3:**
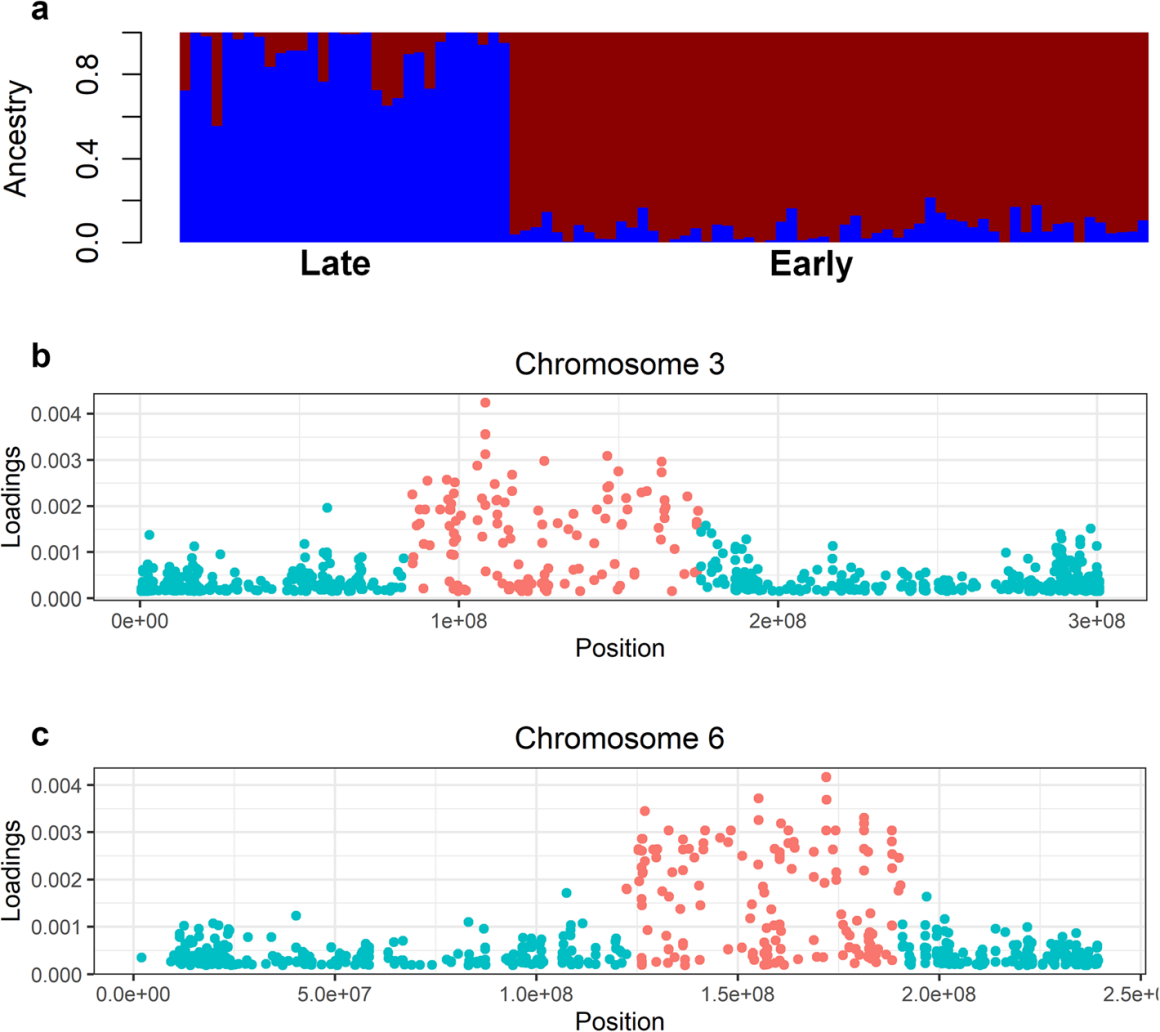
Genome-wide structure analyses of early- and late- flowering morphotypes. **a.** Population structure of the 91 landraces, early-flowering gene pool in red and late-flowering gene pool in blue; **b-c.** Loading plots of the DAPC of early-flowering clusters on linkage groups 3 and 6, respectively

A total of 21,663 filtered high-quality SNPs was identified and used in the diversity study. The data contained an average 3095 SNPs per chromosome and the average of minor allele frequency (MAF) was 0.213. Genome-wide structure analysis revealed two genetic pools that correspond to early- and late-flowering morphotypes (Fig. 3a). Indeed, the sNMF algorithm detected genetic clusters of individuals in the population sample at K = 2 with the lowest cross-entropy value (0.724). Throughout the seven pearl millet linkage groups, sNMF analysis revealed that the lowest cross-entropy values were at K = 2 for LG1 (0.717), LG2 (0.715), LG4 (0.736), LG5 (0.718) and LG7 (0.727). The two K groups corresponded to the two morphotypes. However, LG3 and LG6 are structured in three groups with the lowest cross-entropy value at K = 3 (0.733 and 0.723, respectively): one group representing the late-flowering genetic pool and two others representing clusters of the early-flowering genetic pool (Figure S1). Further, we observed that individuals differentiated at LG3 differ from those differentiated at LG6. The differentiation of the early-flowering millets is linked to LG3 (89.7 Mb) and LG6 (68.1 Mb). This genetic structure was confirmed by DAPC analyses (Fig. 3b-c, Figure S1).

### Identification of SNP markers and candidate genes linked to morphotypes

**Fig. 4:**
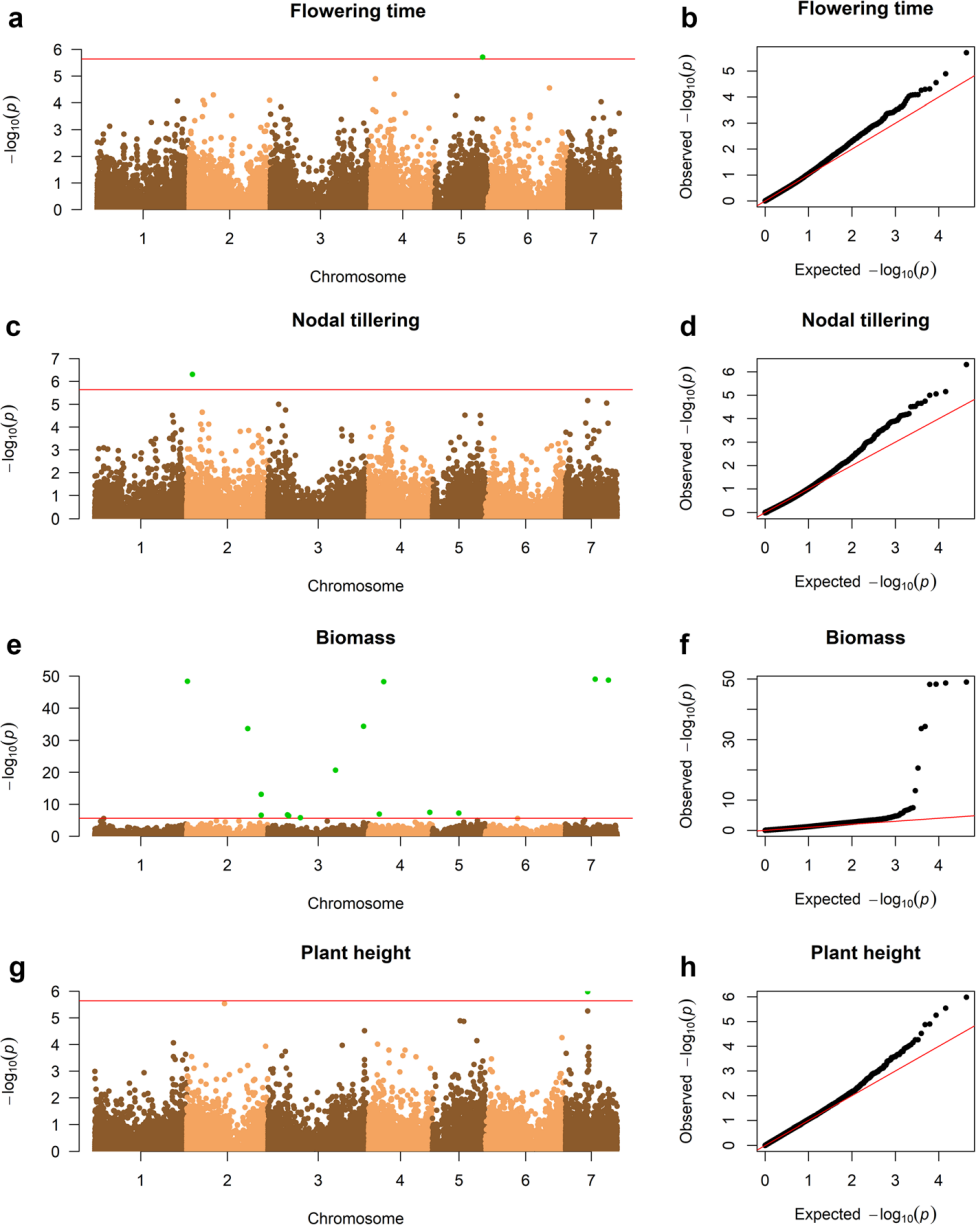
GWAS of early- and late-flowering cultivated pearl millets. Manhattan plots and Q-Q plots for: **a, b.** flowering time; **c, d.** nodal tillering; **e, f.** biomass; and **g, h.** plant height, respectively

The quantile-quantile (Q-Q) plots showed that the mixed linear model (MLM) that accounts for the population structure effect and the kinship matrix was appropriate and had strong statistical power. The GWAS analysis detected 18 SNPs at the threshold of α = 2.3 × 10^− 6^ (Bonferroni correction). These SNPs were associated with biomass, flowering time, plant height and tillering. No SNPs associated with biomass were identified in the peak *P*-value, but they were squatter along the genome at SNPs with very low *P*-values. Flowering time, plant height, and tillering are each tightly associated with a single SNP per trait (Fig. 4, Table S4). For plant height, one SNP is located on the *PgAAO1* gene that encodes an indole-3-acetaldehyde oxidase. For tillering, one SNP is located on the *PgHK4* gene that encodes a histidine kinase. Flowering time is associated with one SNP that is on the *PgPPR* gene that encodes a pentatricopeptide-repeat protein belonging to the ATP DNA-binding cassette family involved in plant resistance and defense.

## Discussion

### Building a core collection of Senegalese pearl millet landraces

Using a heuristic approach with phenotypic and genetic data, we built a core collection of Senegalese pearl millets. This subset of landraces is well distributed across cultivated areas and captures wide genetic diversity and phenotypic variations in the pearl millet morphotypes. The spatial pattern of diversity is correlated with the genetic structure, as previously reported [8] and matches the cultivation areas of morphotypes across the Senegal.

Allogamous species often have fewer redundancies due to gene flow between individuals. The great diversity of pearl millet was associated with dynamic gene flow and admixture between wild and cultivated pearl millet [3]. This is certainly the explanation for the 22% of the core collection we defined, higher than in other self-pollinating species like wheat (*Triticum aestivum*) [14] or rice (*Oryza sativa*) [15]. Our results showed there are no duplicates of landraces in our core collection of Senegalese pearl millet. However, they confirm the previously reported two genetic pools [8–10]: an early-flowering pool subdivided into three clusters differentiated according to yield, spike thickness, tillering, and flag leaf length, and a late-flowering pool differentiated into three clusters according to yield, and tillering traits (Figure S2).

Differentiation of early- and late-flowering morphotypes may have occurred after the domestication of pearl millet (at least 4900 years ago), as a consequence of a center of specialization developing across the Sahel belt. This hypothesis is supported by previous reports of migrations, exchanges and gene flow that led to wider genetic diversity as an adaptive mechanism [3, 4]. These findings and this assumption mean the clusters identified among the early-flowering Souna and late-flowering Sanio are genuine heterotic groups that can be used to breed for earliness and biomass, respectively.

### Phenology and phenotypic traits featuring early- and late-flowering millets

Among the 16 quantitative agro-morphological traits evaluated at many sites and across many years, six were highly discriminating between early- and late-flowering morphotypes with high heritability (*h*^*2*^ ≥ 0.5). There are two phenology features, i.e. heading and flowering time, that are correlated between themselves and also correlated with photoperiod sensitivity in pearl millet. The late-flowering morphotype is more sensitive to photoperiod. The phenology and photoperiod sensitivity are the two mechanisms help pearl millet to adapt to climate variability. Early flowering in pearl millet has been associated with a population adaptation mechanism [7], whereas photoperiod has been considered as an individual adaptation mechanism [5].

In parallel, four traits, biomass yield, plant height, nodal tillering and tillering, associated with the vegetative stage, featured in early- and late-flowering millets with high heritability (*h*^*2*^ ≥ 0.5) (Table 1). Biomass, plant height, and nodal tillering contribute to stover yield, while tillering is also a yield component to maximize grain harvested. This is consistent with observations made by farmers who grow both morphotypes based on their own preferences and agro-systems. For example, some Senegalese farmers located in Niakhar and Bambey villages intercrop both early-flowering Souna and late-flowering Sanio in the rainy season to cope with long dry spells (personal communication).

### Chromosome rearrangements as a source of diversity

Sequencing at the genome-wide scale revealed features at LG3 and LG6 in early-flowering millets, suggesting specific independent rearrangements at these regions for heading and flowering earliness. Chromosomes 3 and 6 might have undergone variations with breakpoints of 89.7 Mb and 68.1 Mb, respectively, large enough to look closely at gene insertion, deletion or synteny (Fig. 3b-c). Indeed, chromosome rearrangements occur as a source of diversity through (i) standing rearrangements that play a role in evolutionary change and adaptive evolution, (ii) rearrangement of transposon elements, is a major mechanism behind the rearrangements we identified, could be catalysts for changes in expression of genes that are altered in association with rearrangements, (iii) variations in transcripts (de novo or level of expression via tandem duplication) [16]. In allogamous crops like pearl millet, extensive chromosomal rearrangements have occurred in its genome since it diverged from a common ancestor with related species [11, 13]. The two later possible sources of variation require more investigation at the gene expression level to test the assumption of independent rearrangements of chromosomes. Rather, we favor the explanation of standing rearrangements, as such variation would provide the genetic diversity needed for a population to rapidly adapt to different environments. This explanation supports evidence for rearrangements in the pearl millet genome revealed by synteny analysis with foxtail millet and sorghum [13].

### Genes underlying allelic and trait diversity

The panel assembled is strongly structured between early-flowering Souna and late-flowering Sanio. Strongly structured diversity between early- and late-flowering millets has been considered to be a way of correcting for population structure and kinship. Correction through structure might be an obstacle to the identification of causal loci that are strongly differentiated between population. However, our results pinpoint some candidates for causal SNPs associated with the measured traits. Consequently, the power of GWAS is mainly identifying SNPs and phenotypic variation within each group. A panel with a more admixed genotype would have been preferable to identify phenotypic differences between early- and late-flowering millets. Biomass led to the identification of a large number of scattered SNPs with no standard peak in the *P*-value around the most significant markers. It is not clear why such a pattern was only observed for biomass. A more classical pattern clearly appeared in other traits, for example, plant height. Correlated genes, *PgAAO* and *PgHK4*, are involved in regulating the plant development response (plant height and nodal tillering) induced by abscisic acid [17] and in the synthesis of phylloquinone that is indispensable for photosynthesis [18], respectively. This suggests that during the vegetative phase, late-flowering Sanio millet produces more tillers, grows taller and captures more light through the hormonal and photosynthesis pathways than early-flowering Souna millet. Allelic variation of these genes might enable these specific phenotypes. On the other hand, during the transition to the flowering phase, the expression of genes may be at the origin of phenotype, as repressors or activators of signaling pathways leading to these features. Orthologs of the *PPR* gene play a role in delaying flowering by mediating the expression of several genes during plant growth, or by repressing genes involved in the transition to the reproductive phase [19]. The *PgPPR* gene we identified could be a putative candidate gene involved in the control of flowering time between early- and late-flowering millets.

### Key features for breeding

The main traits that differentiate the morphotypes in the core collection are yield-related components, biomass, and flowering time. A north-south gradient of early- and late-flowering was identified across the Senegal [8]. There are more early-flowering morphotypes in the north of the country and more late-flowering morphotypes in the south. The special distribution of early- and late-flowering morphotypes also follows a rainfall gradient where the central part of Senegal was on average 500 to 600 mm drier than the south (average 1200 mm) between the 1990s and 2014. Not to mention the fact that certain diseases including downy mildew are more prevalent in the agro-ecological zones that range from the south to the center than from the center toward the north of Senegal [20]. In some areas, early-flowering millet would cope better with drought, while late-flowering millet would adapt to cope with more humid environments. Therefore, harnessing the diversity based on flowering earliness to address climate variability in agro-ecosystems would be a step toward breeding early maturing varieties. Our results showed that the length of the flag leaf and the thickness of the middle axis of the panicles are phenotypes that differentiate the subsets of early-flowering morphotypes with high heritability. These may thus be advantageous traits to target under hotter and drier conditions. The nodal tillering, a character that is consistently associated with fodder yield in pearl millet [21], is also involved in the differentiation of subgroups of both early- and late-flowering morphotypes with high heritability. Based on our results, traits featuring early- or late-flowering millet could be targeted for breeding for dual-purpose varieties (yield and fodder). In summary, most of the characters that differentiate the genetic pools are involved in pearl millet performance in the agro-systems of Senegal.

## Conclusion

The diversity of early- and late-flowering pearl millet landraces from Senegal was captured and a representative core set was defined using an effective heuristic approach. GWAS revealed key phenology and phenotypic traits between the two pearl millet morphotypes while identifying SNPs on genes underlying flowering time, tillering, biomass and plant height. The identification of two subgroups among early-flowering morphotypes on LG3 and LG6 suggests chromosome rearrangements as a source of variation for flowering earliness features. These marker-trait associations could be targeted in breeding programs for pearl millet improvement under erratic climatic conditions.

## Methods

### Defining the core collection and field evaluation

A total of 541 Senegalese pearl millet landraces were collected between 1992 and 2014 [8, 9, 11] from farmers with their approval and respecting institutional, national, or international guidelines. A set of 12 single sequences repeat (SSR) microsatellites [8] were previously used to genotype germplasm consisting of 429 early-flowering morphotypes (Souna) and 112 late-flowering morphotypes (Sanio). In 2014 and 2015, we evaluated 392 landraces at different ISRA’s research stations. A total of 306 early-flowering Souna landraces were evaluated at Bambey (N14°32′12″ W16°36′41″) and at Nioro (N13°45′00″ W15°45′00″), while 86 late-flowering Sanio landraces were evaluated at Senthiou Maleme (N13°49′01″ W13°55′03″) and at Kolda (N12°53′02″ W14°57′05″). In each site, the experiment comprised a randomized complete block design with three replications. Each landrace was grown in a single row containing eight hills. The distance between the rows and between the plants in the row was 90 cm. In the different trials, the following phenotypes were measured: downy mildew incidence (DMI), 50% flowering time (FLO), nodal tillering (NTN), plant height (PHE), tillering (NPT), flag leaf length (FLL), flag leaf width (FLW), spike length (SLE), spike thickness (STH), 1000 seed weight (SWE), grain yield (GYI), panicle yield (PYI) [22]. To establish a core collection from this panel, an advanced maximization sampling technique, called heuristic, was performed based on phenotypic and genotypic data from these 392 landraces using PowerCore v 1.0 software [23]. This algorithm removes duplicates and retains only a limited number of landraces in multiple analyses [23]. From this heuristic approach, 91 landraces were retained, consisting of 60 early-flowering Souna and 31 late-flowering Sanio, that were field-evaluated at ISRA-Nioro in the 2016 and 2017 rainy seasons. The experimental design for each trial was a 7 × 13 alpha lattice with three repetitions. Each of the landraces tested was grown in a single row comprising eight hills in each repetition and the measurements were taken on three hills. The genetic variability of the panel was assessed using the significance of differences between the Nei genetic index of core collection and a Student’s t-test at α = 0.05 [24]. To assess whether the core collection captured the diversity of the whole dataset, we calculated the percentage of mean difference (%MD) and the percentage of variance difference (%VD), the coincidence rate (%CR), and variable rate (%VR) according to [23]. The core collection was considered to be representative of the total collection when no more than 20% of the traits had different means (significant at α = 0.05) in the defined core collection and in the total collected landraces, and the coincidence rate CR% retained by the core collection was no less than 80%. Analysis of variance was performed on the different phenotypic parameters using the Plant Breeding Tools v 1.3 software (https://sites.google.com/a/irri.org/bbi/products) with the formula:

$$ Y=\mu +G+Y+ GY+R+B+\varepsilon $$

where *Y* is the phenotype; μ, the mean; *G* the genetic effect; *Y*, the year effect; *GY*, the interaction between genotype and year; *R* the replication effect; *B*, the incomplete block and *ε*, the residual effect. The heritability of agro-morphological characters was calculated using a mixed linear model with random effects for individuals, using Plant Breeding Tools v 1.3 software (https://sites.google.com/a/irri.org/bbi/products), with the formula:

$$ {h}^2=\frac{\ {\sigma}_G^2}{\left({\sigma}_G^2+\frac{\sigma_{GxY}^2}{y}+\frac{\sigma_{\varepsilon}^2}{ry}\right)} $$

where ${\sigma}_G^2$ is the genotypic variance, ${\sigma}_{GxY}^2$ the genotype by (*y*) year variance and ${\sigma}_{\varepsilon}^2$, the residual variance for (*r*) replicates and (*y*) year.

For each trait, the adjusted mean of each individual in the 2016 and 2017 trials was calculated with fixed-effects for individuals, using Plant Breeding Tools v 1.3 software and was considered as the value of the individual for the trait concerned.

A principal component analysis was performed using the adegenet v 2.1.1 package [25], R v 3.5.1 [26]. A discriminant analysis (DA) between early- and late-flowering millets was then performed and correlation values between phenotypic traits and factual plans were extracted from the DA. Characters that were significant with a *P*-value < 0.001 and which presented a high correlation (r) ≥ 0.7, with the axis of differentiation, were identified as discriminating characters between early- and late-flowering millets. This analysis was performed using XLStat 2014 software (http://www.xlstat.com) and the distribution of discriminating agro-morphological characters was plotted using R software v. 3.5.1 [26]. The distribution of the 91 landraces was mapped using QGIS v 3.8 (https://www.qgis.org).

### DNA extraction, library construction, and sequencing

Genotyping-by-sequencing (GBS) was performed on genomic DNA extracted as previously described [27] from a single plant sampled at the five-leaf stage of each of the 91 landraces grown in 2016 at Nioro. The DNA was checked using a NanoDrop 2000 (Thermo Scientific™) and showed 260/280 and 260/230 ratios between 1.8 and 2, respectively. Extracted DNA was stored in a solution of Tris-HCl and sent for sequencing at the Next Generation Sequencing Platform of the CHU Research Center, University of Laval, Quebec. The libraries were generated in two multiplexes of 45 and 46 samples. *PstI-MspI* double-digestion was applied, and adapters were linked to each sample followed by mixing and amplification. The libraries were sequenced using Illumina HiSeq2500.

### SNP calling, filtering, and data analysis

The quality of the reads was evaluated using FastQC v 0.72 and MultiQC v 1.6, and the sequences were then cleaned with FastQ Trimmer v 1.0.0. Only sequences of average quality (Q) ≥ 30 (Sanger format) were retained and the first 7 bases (5′ side) of each read were removed. The sequences were aligned with the pearl millet reference genome (GenBank Accession number GCA_002174835.2) using BWA v 1.2.3, before realignment of the sequences for insertions and deletions using RealignerTargetCreator v 0.0.4 and IndelAligner v 0.0.6. The binary alignment map (BAM) format files from the above procedures were merged using MergeBAM v 1.2.0 and SNP calling was performed using UnifiedGenotyper v 0.0.6. A total of 545,834 variants were called including 502,382 SNPs. Filtering was first performed based on mapping quality (MQ) and depth, applying hard filtering using VariantFiltration v 0.0.5 (MQ ≥ 40 divided by the depth of unfiltered samples > 0.1). A second filtering was performed according to the minor allele frequency (MAF) > 0.05, and the allowed maximum proportion of missing data was 0.05 for markers and 0.1 for individuals, using Plink v 1.9. Multi-allelic markers were then removed using Tassel v 5.2.48. The output file finally contained 21,663 SNPs and 78 individuals. This dataset was used for all subsequent analyses. All bioinformatics analysis (sequences filtering, cleaning, mapping, and SNPs calling) was carried out on the Galaxy v 18.0.5 platform [28], in the Bio-Linux 8 operating system [29].

### Genetic structure

Genetic structure was evaluated using the sparse mixed linear model (sNMF) algorithm, through the LEA v 2.2.0 package implemented in R. The sNMF algorithm detects genetic clusters of individuals in the population sample. For this analysis, we used several populations ranging from K = 1 to K = 10, with ten repetitions for each K value. Discriminant analysis of principal components (DAPC) was also used through the adegenet v 2.1.1 package [25]. The choice of the number of axes (PCs) retained for the DAPC was made using a cross-validation method, performed on the data set subdivided into two training sets of respectively 90 and 10% [30]. A test comprising 30 repetitions was performed to preselect a limited number of PCs. At each repetition, the validation and training sets were randomly allocated. A second test comprising 1000 repetitions was carried out on the preselected PCs to select the number of PCs that enabled the highest proportion of correct predictions with the lowest error rate. This analysis was carried out using the R software adegenet package [25].

### GWAS

Association analyses were conducted with a mixed linear model (MLM) correcting for population structure and kinship using Tassel v 5.2.48. Q-Q and Manhattan plots illustrating the results of GWAS were produced using the qqman package v 0.1.4 in R [31]. The significance threshold (α) of the association of SNP markers with the different traits was calculated using Bonferroni correction [32]. SNPs significantly associated with agro-morphological traits were localized in the pearl millet genome intervals. Locating was performed with the valR package v 0.5.0 [33] in R. Pearl millet genome annotation was used to identify these genes.

## Supplementary Information

**Additional file 1: Table S1.** List of accessions from the Senegalese pearl millet core collection. **Table S2.** Representativeness statistics of early-flowering Souna morphotype and late-flowering Sanio morphotypes within the core collection. %MD = Percentage of mean difference, %VD = Percentage of variance difference, %CR = Coincidence rate, and %VR = Variable rate. **Table S3.** Means, *P*-values and correlation with axis from the discriminant analysis (DA) of early- and late- flowering millets. **Table S4.** Significantly associated SNPs from GWAS, mapped in the pearl millet genome and associated with phenotypic traits. **Figure S1.** Genetic structure of Senegalese pearl millet landraces core collection at chromosome level (a-g) from chromosome 1 to chromosome 7, respectively. **Figure S2.** Discriminant analysis of (a) early-flowering (Axis1 = 98.49, Axis2 = 1.51) and (b) late-flowering morphotypes (Axis1 = 95.59, Axis2 = 4.41), from phenotypic traits of landraces from the core collection. Boxplots of (c) grain yield, (d) panicle yield, (e) spike thickness, (f) nodal tillering and (g) flag leaf length according to different subsets of early-flowering morphotype. Boxplots of (h) panicle yield, (i) grain yield and (j) nodal tillering according to different subsets of late-flowering morphotype.

**Additional file 2.**

**Additional file 3.**

**Additional file 4.**

**Additional file 5.**

## Data Availability

All the raw sequencing reads for all the landraces are available in additional files. The SNPs generated in this study are included as additional files.

## Abbreviations

GWAS: Genome-wide association study
chr: Chromosome
LG: Linkage group
SNP: Single nucleotide polymorphism
SSR: Simple sequence repeat
GBS: Genotyping-by-sequencing
Mb: Megabytes
Gb: Gigabytes
QTL: Quantitative-trait locus
DAPC: Discriminant analysis of principal components
MLM: Mixed linear model
PCs: Principal components
PCA: Principal component analysis
MAF: Minor alleles frequency
NJ: Neighbor-joining
ISRA: Institut sénégalais de recherches agricoles
sNMF: Sparse non-negative matrix factorization
