# Supplementary material for: GWAS unveils features between early- and late-flowering pearl millets
Source: BMC Genomics. 2020 Nov 10;21:777. doi: 10.1186/s12864-020-07198-2 (PMC7654029; doi:10.1186/s12864-020-07198-2)
Supplement: Supplementary file 1 — Additional file 1: Table S1. List of accessions from the Senegalese pearl millet core collection. Table S2. Representativeness statistics of early-flowering Souna morphotype and late-flowering Sanio morphotypes within the core collection. %MD = Percentage of mean difference, %VD = Percentage of variance difference, %CR = Coincidence rate, and %VR = Variable rate. Table S3. Means, P-values and correlation with axis from the discriminant analysis (DA) of early- and late- flowering millets. Table S4. Significantly associated SNPs from GWAS, mapped in the pearl millet genome and associated with phenotypic traits. Figure S1. Genetic structure of Senegalese pearl millet landraces core collection at chromosome level (a-g) from chromosome 1 to chromosome 7, respectively. Figure S2. Discriminant analysis of (a) early-flowering (Axis1 = 98.49, Axis2 = 1.51) and (b) late-flowering morphotypes (Axis1 = 95.59, Axis2 = 4.41), from phenotypic traits of landraces from the core collection. Boxplots of (c) grain yield, (d) panicle yield, (e) spike thickness, (f) nodal tillering and (g) flag leaf length according to different subsets of early-flowering morphotype. Boxplots of (h) panicle yield, (i) grain yield and (j) nodal tillering according to different subsets of late-flowering morphotype. [file 12864_2020_7198_MOESM1_ESM.docx]

# **Supplementary tables**

Table S1: List of accessions from the Senegalese pearl millet core collection

| Entry | Sample | Morphotype | Phenotypic traits subgroup |
| --- | --- | --- | --- |
| 1 | PgSL1 | Sanio | Subset_1 |
| 4 | PgSL4 | Sanio |  |
| 9 | PgSL9 | Sanio |  |
| 21 | PgSL21 | Sanio |  |
| 23 | PgSL23 | Sanio |  |
| 24 | PgSL24 | Sanio |  |
| 25 | PgSL25 | Sanio |  |
| 26 | PgSL26 | Sanio |  |
| 27 | PgSL27 | Sanio |  |
| 29 | PgSL29 | Sanio |  |
| 31 | PgSL31 | Sanio |  |
| 2 | PgSL2 | Sanio | Subset_2 |
| 3 | PgSL3 | Sanio |  |
| 7 | PgSL7 | Sanio |  |
| 11 | PgSL11 | Sanio |  |
| 12 | PgSL12 | Sanio |  |
| 15 | PgSL15 | Sanio |  |
| 16 | PgSL16 | Sanio |  |
| 20 | PgSL20 | Sanio |  |
| 22 | PgSL22 | Sanio |  |
| 28 | PgSL28 | Sanio |  |
| 30 | PgSL30 | Sanio |  |
| 5 | PgSL5 | Sanio | Subset _3 |
| 6 | PgSL6 | Sanio |  |
| 8 | PgSL8 | Sanio |  |
| 10 | PgSL10 | Sanio |  |
| 13 | PgSL13 | Sanio |  |
| 14 | PgSL14 | Sanio |  |
| 17 | PgSL17 | Sanio |  |
| 18 | PgSL18 | Sanio |  |
| 19 | PgSL19 | Sanio |  |
| 69 | PgSL69 | Souna | Subset _1 |
| 71 | PgSL71 | Souna |  |
| 72 | PgSL72 | Souna |  |
| 73 | PgSL73 | Souna |  |
| 77 | PgSL77 | Souna |  |
| 35 | PgSL35 | Souna |  |
| 38 | PgSL38 | Souna |  |
| 39 | PgSL39 | Souna |  |
| 44 | PgSL44 | Souna |  |
| 48 | PgSL48 | Souna |  |
| 53 | PgSL53 | Souna |  |
| 55 | PgSL55 | Souna |  |
| 59 | PgSL59 | Souna |  |
| 63 | PgSL63 | Souna |  |
| 65 | PgSL65 | Souna | Subset _2 |
| 66 | PgSL66 | Souna |  |
| 67 | PgSL67 | Souna |  |
| 68 | PgSL68 | Souna |  |
| 82 | PgSL82 | Souna |  |
| 83 | PgSL83 | Souna |  |
| 85 | PgSL85 | Souna |  |
| 87 | PgSL87 | Souna |  |
| 88 | PgSL88 | Souna |  |
| 90 | PgSL90 | Souna |  |
| 91 | PgSL91 | Souna |  |
| 70 | PgSL70 | Souna |  |
| 79 | PgSL79 | Souna |  |
| 81 | PgSL81 | Souna |  |
| 32 | PgSL32 | Souna |  |
| 33 | PgSL33 | Souna |  |
| 34 | PgSL34 | Souna |  |
| 36 | PgSL36 | Souna |  |
| 42 | PgSL42 | Souna |  |
| 45 | PgSL45 | Souna |  |
| 46 | PgSL46 | Souna |  |
| 49 | PgSL49 | Souna |  |
| 50 | PgSL50 | Souna |  |
| 51 | PgSL51 | Souna |  |
| 52 | PgSL52 | Souna |  |
| 54 | PgSL54 | Souna |  |
| 56 | PgSL56 | Souna |  |
| 57 | PgSL57 | Souna |  |
| 58 | PgSL58 | Souna |  |
| 61 | PgSL61 | Souna |  |
| 62 | PgSL62 | Souna |  |
| 64 | PgSL64 | Souna |  |
| 84 | PgSL84 | Souna | Subset _3 |
| 86 | PgSL86 | Souna |  |
| 89 | PgSL89 | Souna |  |
| 74 | PgSL74 | Souna |  |
| 75 | PgSL75 | Souna |  |
| 76 | PgSL76 | Souna |  |
| 78 | PgSL78 | Souna |  |
| 80 | PgSL80 | Souna |  |
| 37 | PgSL37 | Souna |  |
| 40 | PgSL40 | Souna |  |
| 41 | PgSL41 | Souna |  |
| 43 | PgSL43 | Souna |  |
| 47 | PgSL47 | Souna |  |
| 60 | PgSL60 | Souna |  |

Table S2: Representativeness statistics of early-flowering Souna morphotype and late-flowering Sanio morphotypes within the core collection. %MD = Percentage of mean difference, %VD = Percentage of variance difference, % CR = Coincidence rate, and %VR = Variable rate

|  | **%CR** |  | **%VR** |  | **%VD** |  | **%MD** |
| --- | --- | --- | --- | --- | --- | --- | --- |
| Souna | 96.97 |  | 131.8 |  | 40.49 |  | 1.7 |
| Sanio | 92.54 |  | 104.58 |  | 14.47 |  | 0.99 |

Table S3: Means, P-values and correlation with axis from the discriminant analysis (DA) of early- and late- flowering millets

| Trait | Early-flowering | Late-flowering | p-value | Correlation |
| --- | --- | --- | --- | --- |
| DM | 0.043 (± 0.039) | 0.004 (± 0.008) | < 0.0001 | -0.5167 |
| HT | 48.378 (± 3.094) | 71.796 (± 5.634) | < 0.0001 | **0.9588** |
| FLO | 52.317 (± 2.897) | 76.801 (± 5.823) | < 0.0001 | **0.9639** |
| NPT | 5.534 (± 0.933) | 7.928 (± 1.012) | < 0.0001 | **0.7835** |
| NTN | 8.207 (± 0.433) | 11.479 (± 1) | < 0.0001 | **0.9375** |
| IL | 20.622 (± 1.046) | 22.025 (± 0.975) | < 0.0001 | 0.5612 |
| FLL | 44.93 (± 3.472) | 48.354 (± 3.229) | < 0.0001 | 0.4448 |
| FLW | 4.641 (± 0.405) | 4.491 (± 0.358) | 0.0858 | -0.185 |
| MSD | 4.232 (± 0.319) | 4.131 (± 0.289) | 0.1413 | -0.1588 |
| PH | 220.266 (± 18.434) | 282.191 (± 18.293) | < 0.0001 | **0.8684** |
| SL | 51.559 (± 7.02) | 43.754 (± 5.702) | < 0.0001 | -0.5034 |
| ST | 8.13 (± 0.714) | 8.993 (± 0.905) | < 0.0001 | 0.4768 |
| PE | 3.956 (± 1.734) | 1.583 (± 1.191) | < 0.0001 | -0.5988 |
| SW | 5.863 (± 0.555) | 5.762 (± 0.478) | 0.3915 | -0.0929 |
| Biomass | 1156.411 (± 318.566) | 2228.964 (± 376.957) | < 0.0001 | **0.8525** |
| GY | 1341.09 (± 418.2) | 2195.8 (± 743.151) | < 0.0001 | 0.6105 |

Table S4: Significantly associated SNPs from GWAS, mapped in the pearl millet genome and associated with phenotypic traits

| **Marker** | **Chromosome** | **Associated phenotypic trait** | **Pvalues** | **FDR** | **Region** | **Distance to the nearest gene (bp)** | **Gene ID** | **Gene name** | **Definition** | **Number of proteins** | **Specific fonctions** | **General fonctions** |
| --- | --- | --- | --- | --- | --- | --- | --- | --- | --- | --- | --- | --- |
| S2_1673638 | 2 | Biomass | 4.52E-49 | 2.94E-45 | Intergenic | 400 | Pgl_GLEAN_10023350 | LTA3 | Dihydrolipoyllysine-residue acetyltransferase component 1 of pyruvate dehydrogenase complex | 3 | Metabolic process, transferase activity, transferring acyl groups | Biological process and molecular function |
| S2_182434549 | 2 | Biomass | 2.01E-34 | 7.25E-31 | Intergenic | 15131 | Pgl_GLEAN_10013800 | MFDR | NADPH:adrenodoxin oxidoreductase | Unknown | Unknown |  |
| S2_222890810 | 2 | Biomass | 2.63E-07 | 0.000437794 | Genic | 0 | Pgl_GLEAN_10027846 | ALDH5F1 | Succinate-semialdehyde dehydrogenase, mitochondrial | 2 | Metabolic process, oxidoreductase activity; oxidation-reduction process | Biological process and molecular function |
| S2_222920292 | 2 | Biomass | 7.61E-14 | 2.06E-10 | Intergenic | -169 | Pgl_GLEAN_10027850 | CCT1 | Choline-phosphate cytidylyltransferase 1 | 1 | Biosynthetic process, nucleotidyltransferase activity | Biological process and molecular function |
| S3_202496771 | 3 | Biomass | 2.22E-21 | 6.86E-18 | Genic | 0 | Pgl_GLEAN_10033123 | Os04g0379900 | Stearoyl-[acyl-carrier-protein] 9-desaturase 5 | 2 | Fatty acid metabolic process, fatty acid biosynthetic process, oxidoreductase activity, acyl-[acyl-carrier-protein] desaturase activity, oxidation-reduction process | Biological process and molecular function |
| S3_286185314 | 3 | Biomass | 3.77E-35 | 1.63E-31 | Intergenic | -657 | Pgl_GLEAN_10023831 | SCAMP6 | Secretory carrier-associated membrane protein 6 | 1 | Protein transport, integral to membrane | Biological process and molecular function |
| S3_58698346 | 3 | Biomass | 2.03E-07 | 0.000366077 | Intergenic | 333 | Pgl_GLEAN_10012689 | PHYLLO | Protein PHYLLO | 4 | Catalytic activity; cellular amino acid catabolic process; thiamine pyrophosphate binding | Biological process and molecular function |
| S3_61879906 | 3 | Biomass | 3.98E-07 | 0.000615194 | Genic | 0 | Pgl_GLEAN_10014062 |  |  | Unknown | Unknown |  |
| S3_97334843 | 3 | Biomass | 1.58E-06 | 0.002279413 | Intergenic | -10696 | Pgl_GLEAN_10000315 | UGT91C1 | UDP-glycosyltransferase 91C1 | 1 | Metabolic process; transferase activity, transferring hexosyl groups | Biological process and molecular function |
| S4_183522008 | 4 | Biomass | 3.45E-08 | 8.30E-05 | Intergenic | 12181 | Pgl_GLEAN_10004825 |  |  | 1 | Unknown |  |
| S4_32445778 | 4 | Biomass | 1.22E-07 | 0.000240007 | Genic | 0 | Pgl_GLEAN_10002428 | ABCF1 | ABC transporter F family member 1 | 3 | Nucleotide binding; ATP binding, ATPase activity, nucleoside-triphosphatase activity | Molecular function |
| S4_45574746 | 4 | Biomass | 5.44E-49 | 2.94E-45 | Genic | 0 | Pgl_GLEAN_10027450 | CPLC4 | Chaperone protein ClpC4 | 7 | Nucleotide binding, DNA binding; nuclease activity, ATP binding Nucleotide-excision repair, Nucleoside-triphosphatase activity | Molecular function and biological process |
| S5_78613055 | 5 | Biomass | 5.56E-08 | 0.000120318 | Intergenic | 17163 | Pgl_GLEAN_10038431 |  | Unknown | Unknown | Unknown |  |
| S7_128335177 | 7 | Biomass | 1.97E-49 | 2.13E-45 | Intergenic | -14 | Pgl_GLEAN_10007334 |  | Unknown | Unknown | Unknown |  |
| S7_88324424 | 7 | Biomass | 9.29E-50 | 2.01E-45 | Genic | 0 | Pgl_GLEAN_10028316 | DDB1B | DNA damage-binding protein 1b | 1 | Nucleic acid binding, Nucleus | Molecular function and cellular component |
| S5_143317884 | 5 | Flowering time | 1.94E-06 | 0.0419816 | Genic | 0 | Pgl_GLEAN_10013349 | PPR | Pentatricopeptide repeat | 1 | Unknown |  |
| S2_16892168 | 2 | Nodal tiller number | 4.91E-07 | 0.01062524 | Genic | 0 | Pgl_GLEAN_10013465 | HK4 | Histidine kinase 4 | 6 | Two-component sensor activity; two-component response regulator activity, two-component signal transduction system (phosphorelay); protein histidine kinase activity, ATP binding, regulation of transcription, DNA-dependent, signal transduction, membrane; phosphorylation, transferase activity, transferring phosphorus-containing groups, peptidyl-histidine phosphorylation | Molecular function, biological process and cellular component |
| S7_66044005 | 7 | Plant height | 1.04E-06 | 0.020333247 | Genic | 0 | Pgl_GLEAN_10031969 | AAO1 | Indole-3-acetaldehyde oxidase | 8 | Catalytic activity; electron carrier activity, oxidoreductase activity, acting on CH-OH group of donors, metal ion binding, flavin adenine dinucleotide binding, iron-sulfur cluster binding, 2 iron, 2 sulfur cluster binding, oxidation-reduction process | Molecular function and biological process |

# **Supplementary figures**


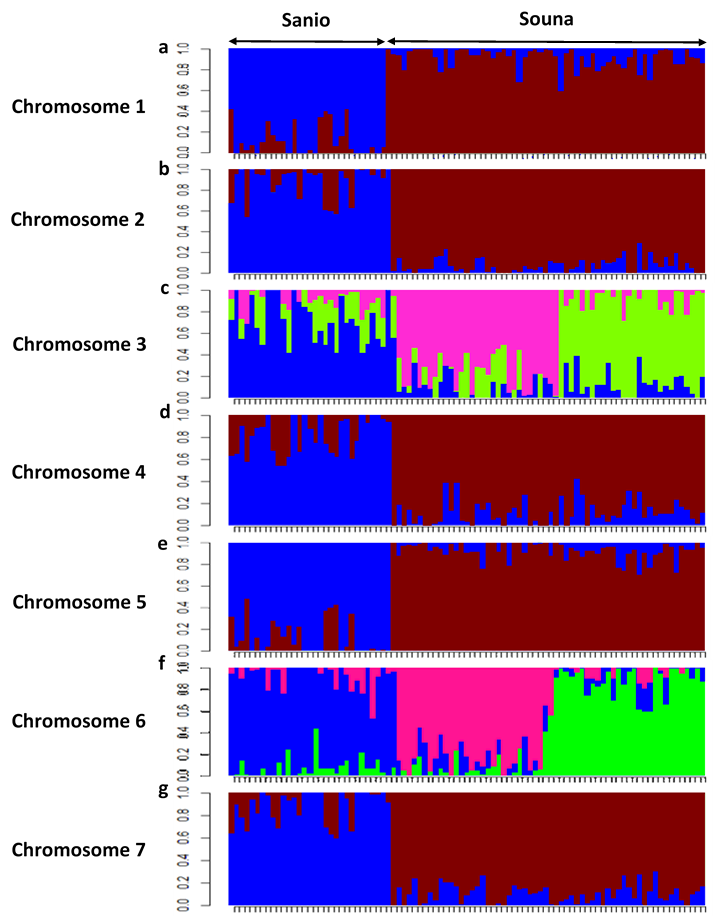


Figure S1: Genetic structure of Senegalese pearl millet landraces core collection at chromosome level (a-g) from chromosome 1 to chromosome 7, respectively


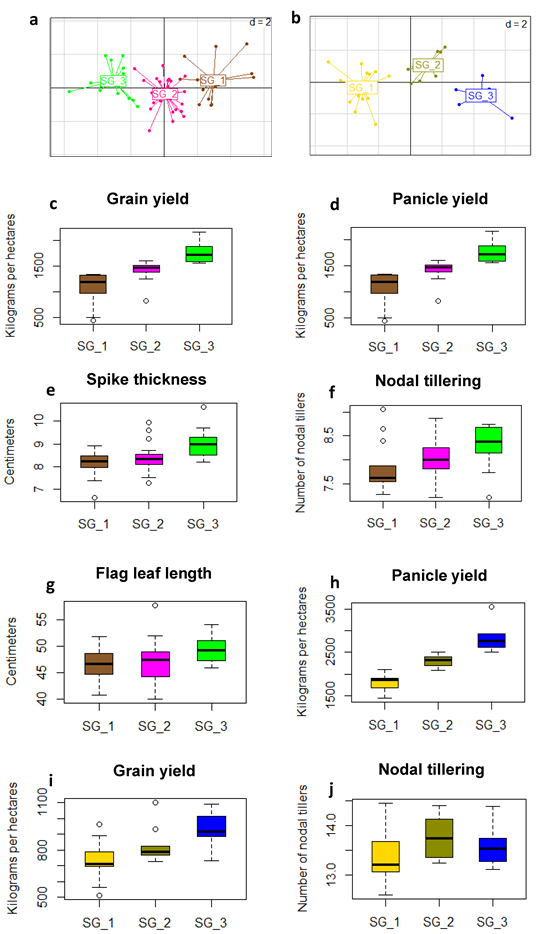


Figure S2: Discriminant analysis of (a) early-flowering (Axis1= 98.49, Axis2= 1.51) and (b) late-flowering morphotypes (Axis1=95.59, Axis2=4.41), from phenotypic traits of landraces from the core collection. Boxplots of (c) grain yield, (d) panicle yield, (e) spike thickness, (f) nodal tillering and (g) flag leaf length according to different subsets of early-flowering morphotype. Boxplots of (h) panicle yield, (i) grain yield and (j) nodal tillering according to different subsets of late-flowering morphotype
